# Canine muscle cell culture and consecutive patch-clamp measurements - a new approach to characterize muscular diseases in dogs

**DOI:** 10.1186/1746-6148-8-227

**Published:** 2012-11-21

**Authors:** Henning Christian Schenk, Klaus Krampfl, Wolfgang Baumgärtner, Andrea Tipold

**Affiliations:** 1 Department of Small Animal Medicine and Surgery, University of Veterinary Medicine, Hannover, Germany; 2 Department of Neurology, School of Medicine, Hannover, Germany; 3 Institute of Pathology, University of Veterinary Medicine, Hannover, Germany; 4 Centre for Systems Neuroscience (ZSN), Hannover, Germany

**Keywords:** Myotubes, Voltage gated ion channels, Functional, Dog, Animal models

## Abstract

**Background:**

The recognition of functional muscular disorders, (e.g. channelopathies like Myotonia) is rising in veterinary neurology. Morphologic (e.g. histology) and even genetic based studies in these diseases are not able to elucidate the functional pathomechanism. As there is a deficit of knowledge and skills considering this special task, the aim of the current pilot study was to develop a canine muscle cell culture system derived from muscle biopsies of healthy client-owned dogs, which allows sampling of the biopsies under working conditions in the daily veterinary practise.

**Results:**

Muscular biopsies from 16 dogs of different age and breed were taken during standard surgical procedures and were stored for one to three days at 4°C in a transport medium in order to simulate shipping conditions. Afterwards biopsies were professionally processed, including harvesting of satellite cells, inducing their proliferation, differentiating them into myotubes and recultivating myotubes after long-term storage in liquid nitrogen. Myogenic origin of cultured cells was determined by immunofluorescence, immunohistology and by their typical morphology after inducing differentiation. Subsequent to the differentiation into myotubes feasibility of patch-clamp recordings of voltage gated ion channels was successfully.

**Conclusion:**

We have developed a canine muscle cell culture system, which allows sampling of biopsies from young and old dogs of different breeds under practical conditions. Patch clamp measurements can be carried out with the cultured myotubes demonstrating potential of these cells as source for functional research.

## Background

In dogs, research into motor unit disorders is a growing field [1-3]. Up to now investigations regarding the pathogenesis are mainly based on electromyography, motor and sensory nerve conduction velocity measurements and histopathological examination of muscle and nerve biopsies or are based on the identification of genetic abnormalities [3-8]. Approaches to evaluate functional components of such disorders in cell culture systems have not been established in veterinary neurology so far.

All previously mentioned procedures for muscle satellite cell culture systems of animals are described predominantly for use under laboratory conditions. Hereby, tissue processing such as harvesting of satellite cells takes place immediately. This limits the application of the technique for investigating spontaneously occurring disease. Only animals kept in the vicinity of or in the laboratory can be used for setting up the primary culture. Furthermore, applying these methods for research into muscle diseases of client-owned animals (dogs, cats) remains questionable. Up to now no procedures have been described to obtain muscle biopsies from healthy or diseased companion animals. Also the influence of shipping to a specialised laboratory for further processing and the impact of long-term storage on cell cultivation and analysis have not been evaluated until now.

Therefore, the aim of the current study was to establish an appropriate shipping method in combination with adequate tissue processing for collecting primary satellite cell cultures in dogs. Furthermore, the feasibility of electrophysiological examinations of voltage- gated ion channels expressed by canine myotubes with the patch clamp technique was evaluated.

## Results

### Cell culture

**Figure 1:**
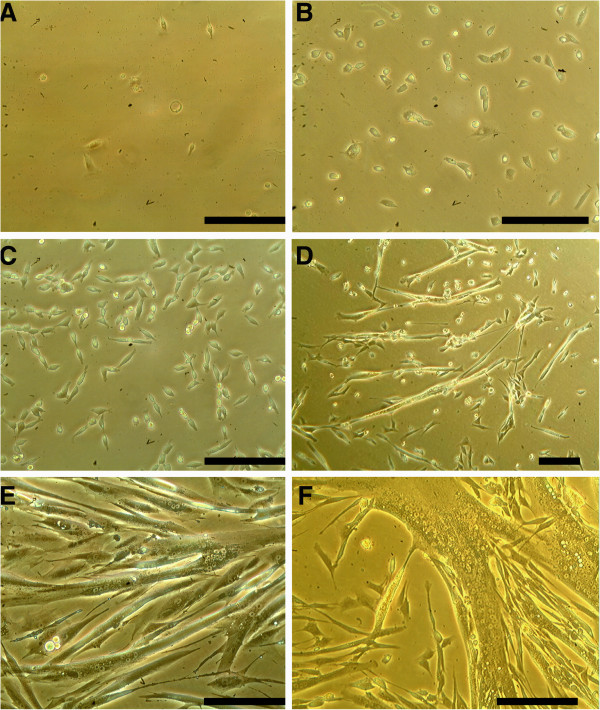
**Photomicrographs of different developmental steps of canine muscle cell culture.A**, **B**, **C** show cells kept under proliferation conditions from 2 to 6 days after harvesting. **D**, **E**, **F** show cells kept under differentiation conditions from 8 to 21 days after harvesting. **A**: a few small blastoid, circular cells are visible. **B** and **C**: typical morphology of proliferating mesenchymal cells. **D** and **E**: fusion of cells and formation of myotubes. At this stage spontaneous contraction of the cells can occur. **F**: finally differentiated multinucleated myotube. (All photomicrographs at 200-fold magnification; except 1D, taken at 100-fold magnification; bar = 100 μm).

**Figure 2:**
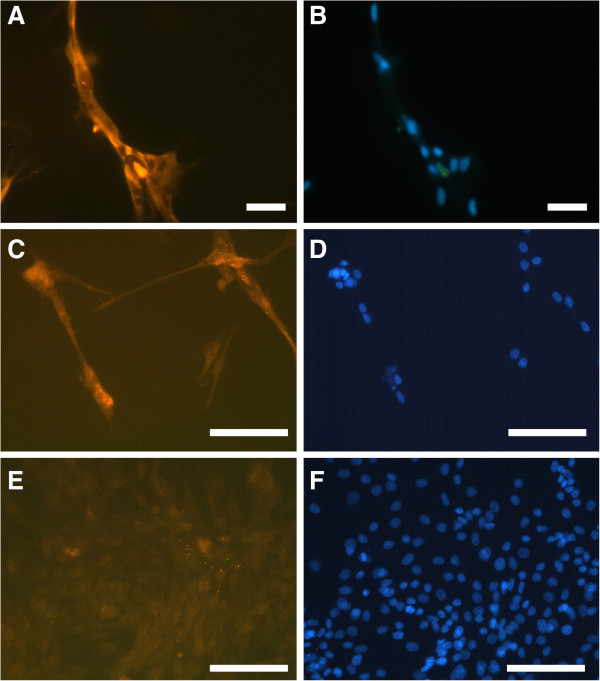
**Immunofluorescence of canine myotubes.A**, **C**: immunofluorescence staining of differentiated, multinucleated canine myotubes positive for muscle-specific intermediate filament desmin, primary antibody: mouse anti-Desmin, secondary antibody: goat anti-mouse IgG, Cy3 labelled. **B**, **D**: 4^′^,6-Diamidino-2-phenylindol nucleus staining (=DAPI) demonstrating multiple nuclei of the myotubes (in 60–80% of the cells). **E**, **F**: canine fibrocytes culture as negative control, (**A**, **B** 400-fold magnification; **C**, **D**, **E**, **F** 200-fold magnification; bar = 100 μm).

**Figure 3:**
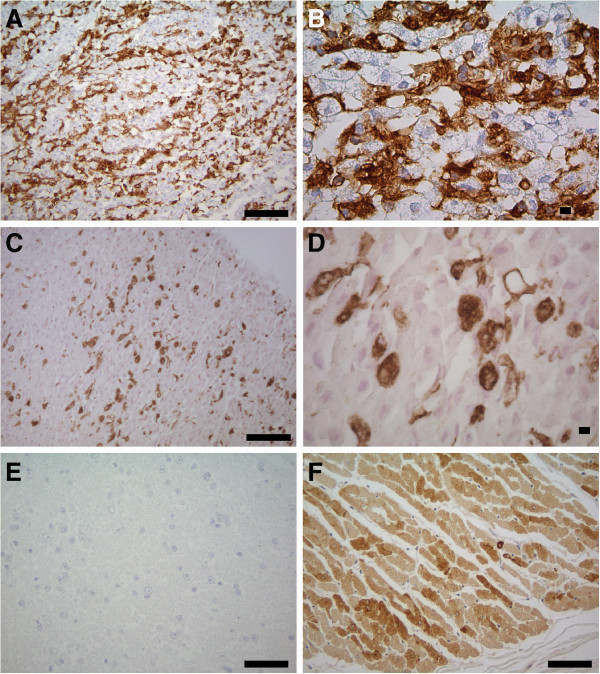
**Immunohistochemistry of cell pellets of myotube cultures.** 3,3^′^ diaminobenzidintetrahydrochlorid (= DAB) stainings of canine myotubes, Primary antibody: mouse anti-Desmin, secondary antibody: goat-anti-mouse-biotinylated, **A**, **B**: (A 100-fold and B 400-fold mag.) primary culture prior to freezing, approximately 70% to 80% of the cells are positive for the muscle-specific intermediate filament desmin. **C**, **D** (C 100-fold and D 400-fold mag.) showing a cell culture pellet after freezing and recultivation. Estimated value of desmin positive cells is 40%. Structural integrity of myotubes is intact, but morphology has been lost due to pellet preparation. **E**: ascites of non-immunised BALB/cJ mice in a dilution of 1:1000 as negative control (100-fold mag.). **F**: normal canine skeletal muscle tissue as positive control, few vimentin-expressing cells (100-fold mag.). Bar= 100 μm. mag. = magnification.

Biopsies were taken during standard surgical procedures from different striated muscle of 16 dogs, stored for one to three days at 4°C in a special transport medium in order to simulate shipping conditions and were further processed (see Table 1). The differential centrifugation steps used to enrich the satellite cells and separate fibroblasts of the currently used method seemed to be the crucial processing steps. Data regarding “id” of the donor, origin of biopsy, age and breed of the animal and further processing of the cultures after the proliferation period of myoblasts are summarised in Table 1. The typical morphology of the muscle cell culture during proliferation and differentiation is demonstrated in Figure 1. Spontaneously occurring contractions of the matured myotubes were documented in video files (Additional file 1: “myotube1.mpg”). These contractions were observed incidentally and no influences on the contraction rate, of age, or breed of donor or duration of storage before processing could be recognized. The initial time necessary for sufficient proliferation varied depending on the age of the biopsy donor. Samples from older dogs (id: 1, 8, 9, 10, 13) needed a longer time period for initiating proliferation (8 to 14 days), whereas samples from younger dogs started to proliferate 2 or 4 days after harvesting. In 6 cases immunofluorescence or immunohistology using a monoclonal antibody directed against the muscle-specific intermediate filament desmin was performed to identify the myotubes (Figures 2 and 3). However, cell morphology was lost due to artifacts obtained during the preparation procedures (e.g. scraping of cells and centrifugation). Therefore, the morphology of the immunohistochemically marked cells (Figure 3) differs in morphology compared to cells labelled by immunofluorescence (Figure 2).

**Table 1:** Data of biopsies processed

**id**	**Age [years]**	** Breed**	** Biopsied muscle**	**Storage time at 4°C prior to processing [d]**	** Processing after proliferation**	**IF**	**IH**
1	9.06	Mix.	M. longissimus dorsi	2	differentiation		
2	0.52	Jack-Russel-Terrier	M. longissimus dorsi	1	contamination		
3	4.93	Mix.	M. rectus abdominis	3	differentiation		
4	0.13	Beagle	M. triceps	2	differentiation		
5	0.14	Labrador	M. biceps femoris	2	freezing, recultivation, differentiation	X	X
6	0.14	Labrador	M. biceps femoris	2	freezing, recultivation, differentiation	X	
7	0.14	Labrador	M. biceps femoris	2	contamination		
8	6.34	Wachtel	M. gluteus medius	1	differentiation		X
9	6.44	Bernese Mountain Dog	M. rectus abdominis	2	differentiation		
10	6.33	Newfoundland	M. gluteus medius	0	differentiation		
11	0.58	Jack-Russel-Terrier	M. gluteus medius	1	differentiation	X	
12	1.34	Mix.	M. gluteus medius	1	differentiation	X	
13	6.46	Mix.	M. rectus abdominis	2	differentiation		
14	0.18	Weimaraner	M. biceps femoris	3	freezing, recultivation, differentiation		
15	7.78	Airedale Terrier	M. intercostalis	0	contamination		
16	3.47	Golden Retriever	M. gluteus medius	2	freezing, recultivation, differentiation		

id = identification number, M. = musculus, d = days, Mix. = mixed breed, IF = Immunofluorescence, IH = Immunohistology, X = indicates preformed staining method.

The cells of the remaining cultures were identified as myotubes due to their typical morphological changes after inducing differentiation. The latter included multinucleated giant cells (over 100 μm; Figures 1D-F). After inducing differentiation cells with fibroblast morphology could be clearly distinguished. The percentage of muscle cells ranged from 60% to 80%. Under differentiation conditions, including achievement of a confluence of up to 80% of the proliferating cells without splitting of the culture, reduction of serum concentration, change to horse serum and withdrawal of growth factors, fibroblasts lacked proliferation. All media used in this study were prepared without any antibiotic-antimycotic supplementation. Only 3 of the 16 cultures had to be discarded due to fungal contamination.

After an initial proliferation period of 8 to 12 days, 4 cultures (id: 5, 6, 14, 16) were frozen at −80°C for 24 hours and then stored in liquid nitrogen for several weeks (up to 8 weeks). Frozen pellets were thawed and recultivated in proliferation medium. In the first 2–4 days of recultivation an increased amount of fibroblast was noticed (Figure 3A compared to 3C). Nevertheless, the amount of the remaining myoblast was between 40% and 60% as shown by morphology and immunohistochemistry (see Figure 3).

In order to simulate shipping conditions the storage period in transport medium for the biopsies prior to processing ranged from 1 to 3 days at 4°C. This time interval allowed for express shipping from various locations of the country to the biopsy processing laboratory.

### Patch-clamp examinations

**Figure 4:**
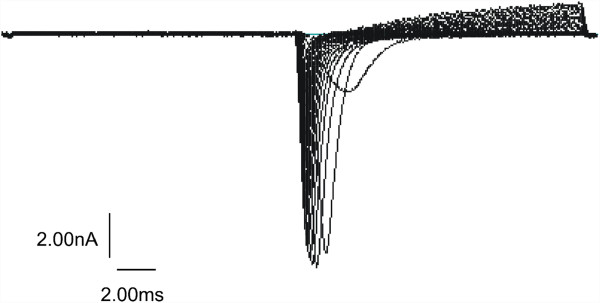
**Whole-cell currents recorded from a canine myotube (mature dog, id 13).** Currents were recorded in response to a series of test potentials ranging from −50 mV to +45 mV in −5 mV steps after an initial hyperpolarisation to −120 mV from a holding potential of −100 mV.

To demonstrate the feasibility of patch-clamp measurements on the cultured canine myotubes, voltage-gated ion channels were evaluated in a pilot study. The applied series of depolarisation pulses initiated whole-cell currents of ion channels in 4 different cells grown out of 3 different muscle biopsies (dog id 5; 8; 13) (Figure 4). By the modalities of the used pulse protocol and the shape of the recorded currents we can state that voltage-gated channels were activated. Due to the small sample size of so far measured cells (n= 4) and the preliminary nature of the electrophysiological study neither evaluation of the kinetics of the recorded data nor the exact characterisation of these channels by different pharmacological tools were performed.

## Discussion

### Cell culture

The feasibility of canine muscle biopsies for muscle cell culture and possible consecutive patch-clamp measurements has been successfully demonstrated and represents an important tool to further investigate functional muscles diseases such as episodic weakness or spasticity and suspected channelopathies in dogs. Furthermore potential animal models for rare human diseases can be identified. Tissue cultures of skeletal muscle satellite cells have been studied in various species (rat, equine, bovine, ovine, dog) [9-15]. Skeletal muscle satellite cells represent a reservoir for prenatal growth of muscle fibres [16]. They are muscular stem cells for growth and regeneration of differentiated muscle tissue and are a source of myogenic precursor cells [17]. These myogenic satellite cells have been the source for muscle tissue cultures. They were harvested from young animals in their activated state as myoblasts and various growth factors have been tested for enhancing proliferation or maintenance in vitro [18-22]. *In vivo* the satellite cells are activated by endogenous factors which are liberated due to postnatal growth or as a consequence of injuries or muscle disease [16,23]. Quiescent satellite cells become activated and proliferate as myoblasts. The proliferation phase of the myogenic cells is terminated by the induction of differentiation. Under in vitro conditions a high local density of myoblasts, a change of serum source and a decrease of serum concentration initiates fusion of the myoblasts to form multinucleated giant cells termed myotubes which finally differentiate to form muscle fibres in the presence of a motor neuron (*in vivo* and *in vitro*) [24-26]. These newly generated fibres fuse with adjacent ones to realise muscle growth or repair damaged muscle tissue *in vivo*[25,27]. The final developmental step of cultured myoblasts, without the presence of a motor neuron *in vitro*, represents the differentiation into myotubes [28,29]. In dogs, methods to establish a primary muscle cell culture under laboratory conditions and to prove the myogenic origin of these cells (most intense staining achieved with desmin D33) have been described [15]. However, procedures have not been established to obtain muscle cell cultures under clinical conditions. Such procedures are used in human muscle disease research to collect samples of rare diseases and for storing the tissue to allow further examinations [30].

In the present study transport of the tissue was simulated by 1 to 3 day storage of the muscle biopsies at 4°C. The applied culturing method proved to be suitable for the purpose of the current study. The method was based on a protocol applied to harvest human satellite cells from muscle biopsies in the Muscle Tissue Culture Collection at the Friedrich-Baur-Institute, Munich. It was modified according to the methods described by Michal et al. [15]. The central point of the modification was the differential centrifugation of the minced and enzymatically dissociated muscle tissue as previously described [22]. During these centrifugational steps the dense fibroblasts and the remaining detritus obtained during mechanical and enzymatic dissociation can be separated from the muscle satellite cells [22]. The differential centrifugation led to an enrichment of muscle satellite cells and a separation of canine fibroblasts. Besides the mechanical removal of all visible connective tissue this is the crucial step for increasing the homogeneity of the cell culture [31].

The applied method for establishing a primary muscle cell culture allowed a successful processing of canine muscle biopsies obtained from healthy animals of different ages and various breeds. Proliferation and differentiation of muscle cells was inducible in all samples. Establishing muscle cell cultures from mature animals is described as being more difficult than from young growing animals [12,32,33]. In the current study the time interval for initiating the proliferation varied depending upon the age of the biopsy donor. However, using the proliferation medium proliferation could be induced in all cultured samples. Potential differences in cell growth considering the different breeds were not examined and are expected to be subtle. Additionally, the donor age might have influence on the ion channel expression of the dogs. Differences between fetal channels and matured channels (e.g. gamma to epsilon switch in the acetylcholine receptor subunits) might occur if neonates are examined. Kinetic patch clamp data were not analysed due to the preliminary nature of the electrophysiological part of the current study. Additionally the cell culture system is as an *ex vivo* technique is still an artificial system using resident stem cells of the muscle. These cells are the origin of cellular growth in the young and in the adult dog. Probably compensating for the age of the donor animal as long as myoblasts can be grown. To verify this hypothesis further patch clamp studies have to be performed.

Disease or injury of muscles may cause a prominent endogenous activation of the satellite cells in living humans and animals [27,34]. Therefore, it is highly probable that muscle cells harvested from diseased animals display enhanced proliferation and differentiation. However, proliferation has to be studied for each disease as shown in dogs with X-linked muscular dystrophy, in which satellite cells were no different in numbers or function from those of wild type dogs, so that at least a comparable amount of cells to the healthy donors in this study can be expected in these cases [35].

An important feature of a cell culture method is the long-term storage of cultured cells in liquid nitrogen and their successful recultivation after thawing [36]. In the current study it could be shown that recultivated canine myoblasts were able to proliferate for at least 3 passages and to differentiate after freezing (up to 8 weeks) and thawing. Continuous cultivation of these primary cell cultures is not possible. The lifespan of all cultured somatic cells is limited to some extent due to the shortening of a deoxyribonucleic acid sequence termed telomere [37]. This problem is also known in human myoblast cultures [38]. To set up a primary cell culture with a longer lifespan further modifications and additional procedures beyond the purpose of the current study would be necessary [39].

The developed method to culture canine muscle cells will enhance the amount and the access of available muscle samples from diseased dogs. Simulation of shipping did not noticeably influence the successful processing of the samples. All previously described methods for primary muscle cell cultures were not applicable for the scientific objectives of clinical muscular research in veterinary neurology [9-14,22]. The developed cultivation method will enable the founding of a muscle tissue bank for samples of various muscle diseases occurring in dogs in different countries providing sufficient material for further in vitro examination methods, without causing unnecessary distress to the diseased animals.

### Patch clamp

Functional studies could be performed using the canine myotubes. The feasibility of patch-clamp measurements has been successfully demonstrated in the second part of this study. Consecutive measurements on the myotubes could identify the ion channel type of the recorded channel (e.g. sodium, potassium) and define their electrophysiological properties. By means of the modalities of the used pulse protocol and the shape of the recorded currents we can conclude that voltage-gated channels were activate. A distinct identification of the type of recorded voltage-gated channels by application of specific blocker substances such as tetrodotoxin has not been performed so far [40]. However, to the authors’ knowledge this is the first described patch-clamp recording of voltage-gated ion channels expressed by canine myotubes. Although a thoroughly electrophysiological evaluation of the expressed channels in normal myotubes still has to be performed, future studies of electrophysiological properties of cells from muscular diseased animals could be compared with those of healthy animals to identify illness underlying pathophysiological mechanisms [24]. This would allow the characterisation of canine skeletal muscle channelopathies, including voltage- and possibly ligand-gated channels or functional deficits in the sarcolemma of inherited myopathies [2,3,41,42]. Another useful application would be the functional characterisation of myogenic cells that carry already genetically identified mutations with an unknown function. Furthermore, new treatment strategies could be developed and tested in vitro using cells from the diseased donors as an alternative to or supplementary to clinical trials [43,44]. Moreover, genetic evaluations of various myopathies could be performed with the myotubes generated using the described cell culture method [45,46]. A therapeutical approach already evaluated for humans using animal models, such as the ex vivo gene manipulation of myogenic stem cells for treatment of myopathies represents another potential future application [47-49]. Myogenic cells collected by the presented culture method could be used for such treatment strategies in dogs.

## Conclusion

The results of the current study offer additional possibilities beside genetic approaches to elucidate functional components of muscular disorders on a molecular level. A collection of biopsies from locations Europe-wide and the foundation of a canine muscle tissue bank are possible using this new technique. Furthermore, functional studies on voltage-gated muscular ion channels with the patch-clamp technique are feasible and can potentially clarify pathomechanisms of so far unknown disease entities of the muscle cell membrane. This also might be the basis for the in vitro development of new treatment strategies.

## Methods

### Cell culture

Biopsies of skeletal muscles from 16 healthy dogs (labelled with identification number (id) 1–16) of various breeds and ages not affected by muscular disease as determined by a detailed clinical investigation (e.g., no clinical signs of a muscle disease, normal blood cell count, normal serum creatinine kinase and lactate levels) were taken during routine surgical procedures such as laminectomy or fracture fixation with the owners’ agreement. All procedures fulfilled the requirements of the German Animal Welfare Act and were approved by the Federal State Office for Consumer Protection and Food Safety of Lower Saxony, Germany (reference number: Az 42502_1). From each dog one biopsy, 1 cm^3^ in size, was taken from different striated skeletal muscles (see Table 1) and transported to the laboratory in 15 mL tubes filled with 10 mL of a sterile transport medium. This transport medium consisted of: 3.6 g N- [2-hydroxyethyle] piperazine-N-2- ethansulphonic acid (HEPES), 3.8 g sodium chloride (NaCl), 0.112 g potassium chloride, 0.99 g glucose and 0.000567 g phenol red dissolved in 500 mL distilled water (aqua dest.). All components were obtained from Sigma-Aldrich. After adding the substances to 400 mL aqua dest. the pH of 7.6 was obtained by using 1 M NaOH. The volume was increased to 500 mL with aqua dest. and the solution was filtered through a 0.22 μm syringe membrane filter (Vivasience AG).

Further processing steps were performed under sterile conditions in a laminar flow bench (CA/R Clean Air). Tissue samples were washed once with fresh transport medium and the adhering connective tissue was removed with forceps and scissors. All biopsies were mechanically minced with scissors to a size of 2 mm^3^. The minced muscle biopsies were digested with a 0.25% trypsin solution. The trypsin-stock-solution (2.5%, Invitrogen) was diluted using the transport medium to a ratio of 1:10. The mechanically dissociated tissue pieces were resuspended in 7 mL of the 0.25% trypsin solution, transferred into a trypsinization flask (Wheaton) and incubated for 7 min. (Shoubridge et al., 1996). During the incubation time the tissue mash was mixed on a magnetic stirrer using a feather-edged magnetic stirring bar. After 7 min. the stirring was stopped to allow the larger tissue pieces to drop to the bottom of the trypsinization flask and the supernatant was poured into a 50 mL tube filled with 10 mL washing medium. The latter was composed of Dulbecco’s modified Eagle Medium (DMEM) (Invitrogen) and 10% Foetal Bovine Serum (FBS) (Invitrogen). 7 mL of 0.25% trypsin solution was added to the remaining tissue mash in the trypsinization flask and stirring and incubation was repeated for 7 min. Thereafter, the supernatant was effused again into a new 50 mL tube filled with washing medium and the whole procedure was repeated. The supernatants of three 50 mL tubes were combined in one tube followed by separation centrifugation as described elsewhere (Greene and Raub, 1992). The remaining pellet was resuspended in proliferation medium and transferred to a 25 cm^2^ tissue culture flask coated with 0.1% PSG. The proliferation medium consisted of Skeletal Muscle Cell Basal Medium (Cat. No. C-23260, PromoCell), Skeletal Muscle Cell Growth Medium Supplement Pack (Cat. No. C-39360, PromoCell), 10% FBS and 1.5% L-alanyl-L-glutamine (Invitrogen).

All cultures were incubated at 37°C in a 5% CO_2_ enriched humid atmosphere and the proliferation medium was changed every other day.

### Freezing and recultivation of myotubes

For freezing and long-term storage the proliferating cells were harvested from the tissue culture flask and resuspended in 1 mL of freezing medium (DMEM, 10% Dimethyl sulfoxide (DMSO), 20% Fetal Calf Serum) at 4°C. Prior to storage in liquid nitrogen the tubes were kept for 24 hours in a −80°C freezer. After several weeks (up to 8 weeks) in liquid nitrogen, the tubes were briefly pre-thawed in a water bath (37.0°C) and with the centre of the pellet still frozen decanted in warm proliferation medium. After 12 hours the proliferation medium was discarded, the cells were washed with PBS and fresh proliferation medium was added.

### Differentiation of myotubes

The mixed cell cultures, consisting of fibroblasts and myoblasts, were kept under proliferation conditions until the cells had reached confluence of up to 80% (approx. 8 to 14 days). In order to induce differentiation of myoblasts into myotubes, cultures with a confluence of up to 80% were not split and proliferation medium was replaced by differentiation medium composed of Skeletal Muscle Cell Basal Medium, 5% horse serum (Invitrogen) and 5 mg Insulin (PromoCell). The different morphological stages of the differentiating cells were documented with an ocular camera (Microscope Systems). The spontaneously occurring contractions of the matured myotubes were filmed as .mpg files using the same camera.

### Immunofluorescence

Cells were fixed with cold (−20°C) methanol and washed with PBS (Biochrom). In order to prevent non-specific binding of the primary antibody 2% of horse serum in PBS was added to the cells overnight at 4°C. Thereafter, cells were washed with PBS before the primary antibody, 1:1 diluted in a PBS /- 0.3% Triton®/- 2% horse serum-solution (anti-Human Desmin Clone D33, Cat. No. M 0760, DakoCytometion) was added. After incubation at room temperature (RT) for 60 min., followed by several washing steps with PBS, the secondary antibody (Cy3- conjugated goat anti mouse IgG, Cat. No. 115165020, Dianova) diluted 1:100 in PBS was added for 60 min. (RT). Cells were washed again three times with PBS prior to nuclear staining with 4′, 6-diamidino-2-phenylindole, dihydrochloride (DAPI, Molecular Probes) at a dilution of 1:20 in PBS and incubated for 4 min. at room temperature (RT). After a final washing step with aqua dest. slides were mounted with 1, 4-Diazabicyclo [2.2.2] octane (DABCO, Sigma). Staining was visualised and photographed with a Leica DMLB microscope, Leica DC 300 camera and Leica software-IM1000 (Leica microsystem AG). Canine fibroblast cultures were used as negative and human myoblast cultures as positive controls for the primary antibodies. Human myoblast cultures were obtained from the Muscle Tissue Culture Collection at the Friedrich-Baur-Institute (Department of Neurology, Ludwig-Maximilians-University, Munich, Germany; part of the German network on muscular dystrophies, MD-NET, service structure S1, 01GM0601; partner of Eurobiobank [30].

### Immunohistochemisty

Cells were removed from the tissue culture flask with a cell scraper (Greiner cell scraper, Sigma) and transferred to an Eppendorf tube and centrifuged by *700* × g for 1 min. Supernatant was discharged and the remaining pellet was fixed with 10% paraformaldehyde (PFA, Sigma) for 24 hours at 4°C. After fixation the cell pellet was embedded in a paraffin wax and 5 μm thick sections were obtained. Sections were mounted on Glass Plus slides (Menzel Gläser), rinsed twice with TRIS-buffered saline (TBS) for 10 min. and blocking of the endogenous peroxidase was performed with 0.03% H_2_O_2_ diluted in TBS for 30 min. Prior to application of the primary antibody, sections were incubated with undiluted goat serum (normal goat serum, Cat. No. S-1000, Vector Laboratories) for 10 min. to block non-specific binding sites. As primary antibody an anti-human desmin monoclonal antibody (clone D33, Cat. No. M 0760, DakoCytometion) was used. The slides were incubated overnight at 4°C with the primary antibody diluted 1:50 in TBS containing 20% of goat serum.

After a washing step with TBS the secondary antibody (biotinylated goat anti-mouse IgG, Cat. No. BA-9200, Vector Laboratories) and the Avidin-Biotin-Complex (Vector Laboratories) was applied for 30 min. (RT). The positive antigen-antibody reaction was visualised by incubating the slides with 3,3′-diaminobenzidine-tetrahydrochloride (DAB)-H_2_O_2_ in 0.1 M imidazole, pH 7.1, for 10 min. (RT) (Sigma). Ascites of non-immunised BALB/cJ mice, diluted 1:1000, were used as negative control for the staining. As positive control normal canine skeletal muscle tissue was used.

### Patch-clamp experiments

For the electrophysiological experiments myotube cultures were transferred into an extra-cellular solution containing 140 mM NaCl, 4 mM KCl, 1 mM MgCl_2_, 2 mM CaCl_2_, 5 mM HEPES, and 5 mM Dextrose. Patch pipettes were pulled from borosilicate glass tubes (GC150TF, Cat. No.: 30–0066, Harvard Apparatus LTD) with a DMZ-Universal Puller (Zeitz Instruments). They had a series resistance between 1.7 and 2 MΩ when filled with an intracellular solution containing 130 mM KCl, 10 mM HEPES, 2 mM MgCl_2_, 5 mM EGTA. The pH of the extra- and intra-cellular solution was adjusted with KOH at 7.4 and the osmolarity to 290 mosmL^-1^ with mannitol.

Only cells morphologically identified as canine myotubes (40 – 60 μm, more than 3 nuclei) were used for patch-clamp experiments. All measurements were performed with an EPC-9 patch-clamp amplifier (List Electronics) and the data were recorded online with HEKA software.

For recording voltage-gated channels in a giga-sealed whole-cell configuration a holding potential of – 100 mV was applied to the myotubes. After hyperpolarisation of the cell to −120 mV (for 15 ms) the first test pulse (15 ms duration) depolarised the membrane to −50 mV. The consecutive series of 19 test pulses of the same duration depolarised the membrane in + 5 mV steps up to + 45 mV.

## Abbreviations

Aqua dest: Distilled water; °C: Degree Celsius; CaCl: Calcium Chloride; Cat. No.: Category number; CO_2_: Carbondioxide, cm^3^, Cubic-centimeter; DMSO: Dimethyl sulfoxide; id: Identification of biopsy donor; DAB: 3,3′-diaminobenzidine-tetrahydrochloride; DABCO: 1, 4-Diazabicyclo [2.2.2] octane; DAPI: 4′, 6-diamidino-2- 327 phenylindole, dihydrochloride; DMEM: Dulbecco’s modified Eagle Medium; FBS: Foetal Bovine Serum; g: Gramm; HEPES: 3.6 g N- [2-hydroxyethyle] piperazine-N-2- ethansulphonic acid; H_2_O_2_: Hydrogenperoxide; KCl: Potassium Chloride; KOH: Potassium hydroxide; mm^3^: Cubic-millimetres; MgCl_2_: Magnesium Chloride; MΩ: Mega Ohm; mL: Millilitre; mM: Millimolar; mosmL: Milliosmol; mV: Millivolt; μm: Micrometers; min.: Minutes; NaCl: Sodium Chloride; NaOH: Sodium hydroxide; PSG: Porcine Skin Gelatine, PFA, Paraformaldehyde; PBS: Phosphate Buffered Saline, RT, Room Temperature; TBS: TRIS-buffered saline.

## Competing interests

None of the authors of this paper has a financial or personal relationship with other people or organisations that could inappropriately influence or bias the content of the paper.

## Authors’ contributions

HCS developed the study idea/ design, performed the whole study and wrote the manuscript. KK contributed to the study design and advised for the Patch Clamp experiments. WB assisted for the immunohistological examinations and advised in the cell culture development. AT developed the study idea/ design, advised HCS during the whole accomplishment of the study and critically revised the manuscript. All authors read and approved the final manuscript.

## Supplementary Material

Additional file 1 Supplementary video file showing spontaneous contractions of a myotube in the cell culture can be found in the online version, at doi: XYZ. Click here for file
